# Improving equity through barrier-free transportation: an evaluation of Shanghai metro stations

**DOI:** 10.3389/fpubh.2024.1463824

**Published:** 2025-01-16

**Authors:** Zhan Zhang, Xiongjie Yang, Chenming Jiang, Linjun Lu, Xiaoyue Li, Xiaoyu Rong, Ziqi Huang

**Affiliations:** ^1^School of Design, Shanghai Jiao Tong University, Shanghai, China; ^2^China Institute of FTZ Supply Chain, Shanghai Maritime University, Shanghai, China; ^3^School of Ocean and Civil Engineering, Shanghai Jiao Tong University, Shanghai, China

**Keywords:** barrier-free design, equity evaluation, analytic hierarchy process, Shanghai, metro stations inclusive urban mobility, transit-oriented development (TOD), accessibility, analytic hierarchy process (AHP)

## Abstract

**Introduction:**

As urbanization progresses and vulnerable populations increase, equitable accessibility remains a critical issue. This study evaluates the accessibility of transit-oriented development (TOD) in Shanghai, focusing on barrier-free facilities in metro stations.

**Methods:**

A comprehensive evaluation framework combining the Analytic Hierarchy Process (AHP) and the System Usability Scale (SUS) was developed to assess metro station accessibility. Thirteen evaluation factors formed a composite accessibility index. A case study of two Shanghai metro stations, Xinzhuang and Xujiahui, was conducted using quantitative metrics, surveys, and interviews.

**Results:**

A strong correlation between AHP scores and SUS ratings validated the framework’s reliability. The study provides recommendations for enhancing metro accessibility.

**Discussion:**

The proposed framework offers a robust tool for evaluating and improving urban transit accessibility, with implications for inclusive mobility policy and design.

## 1 Introduction

“Transit-Oriented Development” (TOD) is a community planning approach that aims to prevent urban sprawl and improve public transport accessibility, creating livelier neighborhoods (1). Under the guidance of public transportation, TOD aims to create compact, mixed-use urban spaces with pedestrian-friendly environments. This approach enhances the efficiency of public transit use while fostering vibrancy and attractiveness in communities along transit corridors (2). The public transportation accessibility mentioned refers to people’s opportunities to access and use public transit services (3), providing various populations with the means to obtain urban services.

It is widely acknowledged that physical design is crucial to the success of TOD. Among the elements involved, auxiliary service systems, aside from transportation infrastructure, serve as the ‘user interface’ between TOD and its users. Tracing the development of TOD research, inclusivity was embedded in the original concept of TOD, emphasizing affordability (by fully reducing housing and transportation costs) and improving accessibility for vulnerable groups, such as children and the older adult (4). However, despite the inherently ‘moral’ and ‘social’ qualities of TOD, the high economic benefits associated with its implementation have led research to increasingly focus on issues of land use, transportation, and planning management, with limited attention to inclusivity for disadvantaged groups and even less to accessible auxiliary service systems (5, 6). The complex road environments and spatial layouts within TODs often result in ‘design exclusion’ for people with disabilities, adversely affecting their experience and limiting their willingness to travel (7). Without appropriate strategies to support inclusivity, people with disabilities and the older adult may face social and digital exclusion within smart cities.

Mobility impairment is a category of disability that encompasses individuals with various physical disabilities resulting from illness, injury, age-related conditions, and other factors. People with mobility impairments often rely on assistive devices or mobility aids, such as canes, crutches, wheelchairs, and prosthetic limbs, to facilitate movement, which can make accessing conventional forms of transportation challenging, particularly in areas with steep inclines. To improve accessibility for individuals with mobility impairments, while also considering the construction and financial constraints of infrastructure projects (8), authorities could consider offering specialized paratransit services that would either fully or partially meet their needs (9).

Originating from the humanitarian ideas of the early 20th century, the barrier-free design has emerged as a new approach in architectural design. Since the 1960s, the concept of barrier-free design has spread from the Nordic countries to other parts of the world (10). Following the United Nations’ formal introduction of the concept of “barrier-free design” in 1974, China began to advocate for the construction of barrier-free facilities and environments in 1985 (11). In 2022, China’s ‘14th Five-Year Implementation Plan for Accessible Environment Construction’ was introduced, calling for the accelerated integration of digitalization with accessible environments and designating information accessibility as an essential component of new smart city and digital countryside development (12). In 2022, Beijing issued the ‘Guidelines for Systematic Accessible Design in Beijing,’ which represents the most advanced accessibility standards in China to date, with a dedicated chapter addressing regulations for transit stations (13). The Winter Paralympics further refined technical standards, providing detailed specifications in areas such as general accessibility, accessible transportation, accessible facilities, accessible accommodation, and other criteria, thus establishing clear guidelines for construction (14). However, there remains a significant gap in research on TOD that considers transit behaviors and human-centered accessible facilities (15). This study, therefore, takes Shanghai as a case study to conduct an evaluative analysis of barrier-free facilities within TODs.

## 2 Literature review

Accessible public spaces are essential for fostering inclusive communities that accommodate diverse needs across various demographics, including age, gender, language, and physical abilities. Existing research on accessibility estimation methods in public space design has identified several key approaches, each with distinct advantages and limitations. These include the qualitative method, user research method, quantitative approach, machine learning method, and the spatial mapping and simulation method, the latter of which has emerged as a valuable addition to the estimation toolkit in recent years.

Qualitative analysis of accessibility could contribute to a deeper understanding of user behavior and accessibility requirements in diverse public settings. By evaluating the accessibility of public space through manual observation and empirical judgment, researchers got a close look to the users. Han et al. (16) conducted a systematic review of behavior studies within open public spaces, noting that qualitative methods are instrumental in capturing nuanced interactions and spatial usage patterns, which are often overlooked in quantitative assessments alone. Phaholthep et al. evaluate Narasuwan University Hospital’s public space using a universal design-based method to assess its adherence to the seven principles for accommodating the requirements and limitations of vulnerable groups (17). Baghernejad Hamzehkolaee (18) applied the spatial syntax method to quantitatively analyze qualitative factors within public spaces, underscores the value of integrating spatial configuration assessments to enhance the understanding of accessibility levels, particularly in complex environments like public parks and historical sites. Some studies have utilized empirical latent variables (19), while others have relied on established psychological models of individual decision-making, such as the Theory of Planned Behavior (20), to examine intentions around using alternative transportation options (21). Sturge et al. (22) explored the use of qualitative methods to assess the needs and resources for individuals with memory problems and dementia in public spaces. Despite the inherent challenges of subjectivity and limited reproducibility associated with qualitative methods, these studies affirm their indispensable role in uncovering user-centric perspectives, making them essential complements to quantitative assessments in comprehensive accessibility evaluation frameworks.

Gamache et al. (23) examed accessible pedestrian infrastructures literatures via mapping review and found out few studies examine the same design to determine the extent to which it aids, hinders, or is neutral (neither helping nor hindering) for people with different disabilities. Since what one person perceives as an aid might be an obstacle for another, it is crucial to compare the usefulness of design features across different groups to identify solutions that work for the majority of users. This can be achieved not only through product- or technology-oriented research, such as testing specific materials or configurations, but also through user-centered research that identifies the needs of individuals by collaboratively developing infrastructure with users from the outset. Therefore we introduced user study into this context. Therefore we introduces user-centered study method and use SUS to verify.

User-centered research is the systematic study of target users, employing a variety of methods to identify issues and design opportunities, and to ascertain pertinent information that can be utilized in the design process (23). Such methods include the collection of feedback from individuals with disabilities through the use of surveys, interviews, and focus groups, with the objective of understanding their needs and experiences. To illustrate, Kosara et al. (24) sought to develop inclusive design guidelines based on a user study that included surveys and observations of 100 individuals with diverse disabilities and abilities in a park setting. Arora et al. (25) employed a mixed-methods approach, integrating questionnaires and observations, to facilitate the creation of more inclusive and accessible public spaces by designers. Pierard and Lee (26) used observation and flipchart surveys to collect data and immediate feedback on library user behavior as a basis for optimizing the design of accessible spaces. From the perspective of user-centered study, its limitations are primarily evident in the sacrifice of a comprehensive exploration of a multitude of topics in the pursuit of an in-depth analysis of specific issues, which consequently affects the comprehensiveness of the results.

Some quantitative approachs employ measurable indicators and statistical analysis, enabling the identification of the most effective features for enhancing the accessibility of public spaces. Mehta (27) discussed the evaluation of public space and developed a public space index based on five criteria. Mosca et al. proposed an evaluation framework based on Universal Design (UD) principles, aiming to improve the accessibility and adaptability of buildings for different user groups (28). Study (29) utilized automatic fare collection (AFC) data to examine the usage of accessible elevators at metro stations and proposed an evaluation system based on supply types and facility layout. Researchers in the study (30) analyzed transportation, pedestrian-oriented accessibility, and urban development dimensions, identifying 12 indicators that were categorized into five clusters. Some researchers (31, 32) use machine learning, which improves model prediction accuracy by automatically learning and refining the model. Liu et al. (31) proposed a model based on geographic information and environmental data that can accurately guide the construction of public accessibility facilities for the disabled and older adult. Xue et al. (32) used clustering algorithms and geometric center methods to optimize the deployment of barrier-free service resources in airports for passengers with disabilities.

In the pursuit of assessing the accessibility and quality of urban environments, certain investigators have recourse to quantifiable indices (33). Within the purview of urban open space evaluation, the Analytic Hierarchy Process (AHP) has been useful in determining the relative significance of diverse factors that underpin the quality and accessibility of public spaces (34). Shi et al. (35) used AHP and DEA models, selected evaluation indicators based on multi-source data, quantifies Hangzhou citizens’ preferences for the functional, aesthetic, and environmental attributes of open spaces for daily use, which directly influences the consideration of public space design and accessibility in urban planning. Saaty et al. (36), view AHP as a decision making tool to structure hierarchical model incorporating the expertise and empirical knowledge of decision-makers. In this domain, discerning the relative significance of diverse amenities and design elements poses a significant challenge. AHP surmounts this challenge by employing pairwise comparisons to elucidate the quantitative relationships between elements at analogous hierarchical levels and those at superior levels, thereby determining the relative weight of each factor.

The user research qualitative method, though easy to use, faces challenges such as weak objectivity, limited reproducibility, comparability, and subjective biases (37). The machine learning method needs a large number of high-quality samples, which are very difficult to obtain in grand scenes such as TOD. In addition, this method is currently poorly interpretable and unable to provide relevant suggestions. The quantitative approach constructs more precise criteria, leading to a more comprehensive and rigorous evaluation system. However, studies that adopted this method still have some limitations: (I) insufficient experimental verification, (II) mostly set facilities as indicators without clear criteria for these facilities per se, (III) have not taken TOD into account.

Therefore, by integrating theoretical and practical perspectives, employing data analysis techniques, and applying analytical methods to user-centered research data, we try to offers quantitative evidence to support theoretical propositions and provides valuable insights for the theoretical advancement and practical implementation of accessible design.

## 3 Structure of evaluation framework

This study used a mixed-methods approach, combining qualitative and quantitative methods. First, a set of key factors affecting the accessibility of vulnerable groups in TOD scenarios was identified via official critera and literatures. Second, a survey questionnaire was designed and administered to collect data on the accessibility of selected TOD scenarios in China. Third, field visits will be conducted to selected TOD scenarios to observe the physical environment and evaluate the accessibility of barrier-free facilities. Finally, data analysis will be conducted to identify the strengths and weaknesses of accessibility in TOD scenarios and provide implications.

### 3.1 Developing evaluation indicators

Through analysis of field studies and the Chinese government’s regulation concerning barrier-free design (GB50763-2012) (38), a set of evaluation criteria has been identified to represent the barrier-free system of TOD. As is represented in Table 1, there exist 13 selected indicators, classified into four main aspects: the first layer is the barrier-free evaluation of the TOD and serves as the goal of the entire AHP. The second layer consists of functionality, accessibility, continuousness, and matching degree. The third layer features a barrier-free elevator, escalator, barrier-free stairs and steps, wheelchair ramp, tactile paving, barrier-free parking lot, low service facilities, barrier-free toilet, turnstile, visual guide, braille, voice prompt, and manual service.

**Table 1:** The barrier-free system of TOD.

Category	Evaluation factors	Explanation
Functionality	Barrier-free elevator	A type of elevator designed to provide accessibility for individuals with disabilities
	Escalator	A moving staircase that carries people between different levels of a building.
	Barrier-free stairs and steps	Structures that are designed to ensure safe and easy access for all individuals, by incorporating features such as gradual slopes, handrails, and non-slip surfaces
	Wheelchair ramp	An inclined plane designed to allow wheelchair users to access buildings or areas with height differences.
	Tactile paving	A surface treatment designed to assist visually impaired and blind pedestrians by providing a tactile and visual contrast to indicate the direction.
	Barrier-free parking lot	A designated parking area specifically designed to provide easy access and convenience for individuals with disabilities.
	Low-service facilities	A reception desk that is designed to be at a lower height than a standard desk. A ticket vending machine
	Barrier-free toilet	A restroom facility that is designed to accommodate people with disabilities, providing features such as grab bars, larger stalls, and enough space for wheelchair maneuvering.
	Turnstile	A mechanical gate used for crowd control or to restrict access to a specific area, which typically rotates horizontally or vertically and allows only one person to pass through at a time.
	Visual guide	A tool or resource, such as pictures, diagrams, or videos, that provides information or instructions in a visual format
	Braille	A system of raised dots that can be felt with the fingertips and is used by people who are blind or visually impaired
	Voice prompt	An audio message played by a device to guide a user through a series of actions or provide information
	Manual service	A type of service in which human labor is required to perform tasks
Accessibility	/	The degree to which a facility is easily accessible in terms of spatial location.
Continuousness	/	The degree of continuity set in the spatial location, and whether there are pain points in the user’s experience.
Matching degree	/	The matching relationship between the demand and supply of a facility

### 3.2 Factor quantification

This section is going to give a detailed introduction to the quantification process of these 13 selected factors. Functional formulas primarily relate to the dimensional requirements of the hardware facilities themselves, as extracted from national standards. However, some other aspects involve subjective evaluations. For instance, the formula for barrier-free elevator and lifting platform consists of two parts:

#### 3.2.1 Physically


(1) $$\left(\frac{d}{1.4},1\right)\min*\left(\frac{w}{1.1},1\right)\min*\left(\frac{d2}{1.5},1\right)\min*\left(\frac{w2}{0.8},1\right)\min$$

Equation 1 shows the formula for calculating the score in terms of the physical attribute of barrier-free elevator and lifting platform. The *d* is the depth of the cargo, w is the width of the cargo, *d2* is the depth of the waiting hall, and *w2* is the width of the door. The unit is a meter. Additionally, considering the need to constrain the results of the indicators within the range of 0 to 1, the introduction of the minimum function is necessary.

#### 3.2.2 Subjectively

There are blind roads and barrier-free signs at the entrance and exit, marked as 0 or 1. The overall core for this index is the average of physical and subjective scores. They got equal weight. If the station did not have any of this type of facility, the indicator was marked 0.

#### 3.2.3 Functionality

Functionality refers to the use of the functionality of the facility itself. Table 2 shows the evaluation criteria.

**Table 2:** The evaluation criteria.

Factors	Key dimensions	Evaluation criteria
Barrier-free elevator	Internal space of cargo, depth of waiting hall, size of door	$\left(\frac{d}{1.4},1\right)\min*\left(\frac{w}{1.1},1\right)\min*\left(\frac{d2}{1.5},1\right)\min*\left(\frac{w2}{0.8},1\right)\min$ The *d* is the depth of the cargo, *w* is the width of the cargo, *d2* is the depth of the waiting hall, and *w2* is the width of the door. The minimum function makes the result between 0–1. The unit is a meter. There are blind roads and barrier-free signs at the entrance and exit, marked as 0 or 1.
	Blind roads and barrier-free signs at the entrance/exit	Mark as 0 or 1
Escalator	Easy access, voice broadcast	Mark as 0 or 1
Barrier-free stairs and steps	Non-slip tread, obviously layered steps	Mark as 0 or 1
	Reasonable step size	$\left(\frac{160}{h},1\right)\min*\left(\frac{w}{280},1\right)\min$ *H* is the height of the step; *w* is the width of the step. The unit is a millimeter.
Tactile paving	Distinguished colors, non-slip profile with reasonable grain	Mark as 0 or 1
Barrier-free parking lot	Flat, non-slip surface with parking lines and wheelchair access lines, and barrier-free signs	Mark positions closer to entrances/exits as 0 or 1
	Wheelchair ramp with reasonable size and slope	Score calculation: if (1:20 < slope < 1:8), score = 8.333*slope-0.042; else score = 0; *(width/1) min
	Safety blocking measures, smooth and non-slip slope, non-reflective surface, and barrier-free signs	Mark as 0 or 1
Low service facilities	Reasonable height and size of lower space	$\left(1,\frac{700}{H}\right)\min*\left(1,\frac{W}{750}\right)\min*\left(1,\frac{H1}{650}\right)\min*\left(1,\frac{D}{450}\right)\min$ The *H* is the height of the upper surface of low-level service facilities from the ground. The *W*, *H*1, and *D* are, respectively, the width, height, and depth of the moving space for the knees and toes of wheelchair users at the lower part of the low-level service facilities. The unit is a millimeter.
	Wheelchair swing space in front of a desk	$\left(\frac{\mathrm{diameter}}{1.5},1\right)\min$
Barrier-free toilet	Marked entrance and non-slip, no water accumulation on the ground	Mark as 0 or 1
	Reasonable doorway size and internal space	$\left(\frac{d}{800},1\right)\min*\left(1,\frac{L}{2.00}\right)\min*\left(1,\frac{W}{1.50}\right)\min*\left(1,\frac{L}{1.80}\right)\min\mathrm{or}\;\left(1,\frac{W1}{1.00}\right)\min*\left(1,\frac{C}{1.50}\right)\min*\left(1,\frac{D}{800}\right)\min$
Turnstile	Reasonable traffic width	$\left(\frac{W}{0.9},1\right)\min$
	The convenience of card swiping	Mark as 0 or 1
Barrier-free parking lot	Flat, non-slip surface with parking lines wheelchair access lines, and barrier-free signs	Mark positions closer to entrances/exits as 0 or 1
	Wheelchair ramp with reasonable size and slope	If (1:20 < slope < 1:8), score = 8.333*slope-0.042; else score = 0; *(width/1) min
	Safety blocking measures, smooth and non-slip slope, non-reflective surface, and barrier-free signs	Mark as 0 or 1
Visual guide	Eye-catching, readable, clearly indicates the direction/location of barrier-free facilities and access mode/formation system	Score from 1–3
Braille	Reasonable content and obvious location, protrusion, and readability	Score from 1–3
Voice prompt	Clear broadcast sound with reasonable content	Score from 1–3
Manual service	Enthusiastic and patient attitude, timely and professional service, able to solve travel problems for vulnerable groups	Score from 1–3

#### 3.2.4 Accessibility

Accessibility refers to the degree to which a facility is easily accessible in terms of spatial location. The construction of this factor was based on graph theory, which was first applied to studies on transportation in the 1960s by Lehman (39). The first step involves identifying the barrier-free facilities as nodes and representing the connections between them as edges in the graph. Each node represents a specific facility, and the edges symbolize the potential pathways between the facilities. During the graph formation process, obstacles such as walls are taken into account. When a potential connection encounters an obstacle, it is canceled, ensuring that the graph accurately reflects the physical interconnectivity of the facilities. Once the graph is constructed, the network depth is calculated as the average shortest path length between all pairs of nodes. This involves finding the shortest path between every possible pair of nodes using an appropriate algorithm (e.g., Dijkstra’s algorithm) and calculating the average length of these paths.

The calculated network depth value provides valuable insights into the level of interconnectivity within the graph. A lower network depth indicates a higher degree of connectivity, suggesting that the barrier-free facilities are more closely connected. This implies that individuals with disabilities can navigate the environment more efficiently, reducing travel distances and potential barriers.

#### 3.2.5 Continuity

Continuity refers to the degree of continuity set of tactile paving in the spatial location, and whether there are pain points in the user’s experience.


(2) $$FAM=\frac{\alpha *Dis*Naf}{\mathrm{Ndic}*Nf*Nch}$$

Equation 2 shows the formula for calculating continuity, in which *Naf* represents the total count of facilities, *Nf* represents the count of facilities that are connected to the tactile paving, and *Nch* represents the count of changes in directions of the tactile paving. *Dis* denotes the total length of the tactile paving, and *Ndic* corresponds to the number of points where the tactile paving is discontinuous. Additionally, we have the adjustment factor *α*, which is used for calculation purposes. Here the alpha is 0.1 in this project.

#### 3.2.6 Matching degree

Matching refers to the matching relationship between the demand and supply of a facility. The method for calculating spatial compatibility involves determining the effective service range by drawing circles around each accessible service facility and performing Boolean operations (40) on the resulting circles. The calculation involves determining the total area of service facility coverage and comparing it to the overall planar area of the TOD site. Overlapping areas between circles are not double-counted. Essentially, this method is based on the top-down view of object shapes. Equation 3 shows the formula for calculating matching degree.


(3) $$\mathrm{Accessibility}\;\mathrm{Ratio}=\frac{\mathrm{Stotal}}{\mathrm{Saccessible}}*100\%$$

Stotoal stands for the while Saceesible stands for the service area.

### 3.3 Factor normalization

The previous analysis reveals the assignment of distinct quantitative scores to various evaluation factors. To consolidate these factors into a composite index, it becomes crucial to normalize their original values, a procedure commonly known as factor normalization. In this paper, we adopt the factor normalization formula proposed by Krajnc and Glavič (41) as the chosen approach for this purpose (as shown in Equation 4).


(4) Sn=Sa−Smin/Smax−Smin

The normalized score (SN) is derived from the actual score (SA), where Smax and Smin represent the maximum and minimum achievable scores, respectively. In this study, it is important to note that the minimum score for every evaluation factor is consistently zero. As a result, the equation can be redefined accordingly (as shown in Equation 5).


(5) $$Sn=\frac{Sa}{\mathrm{Smax}}*100\%$$

Following this process, the quantitative score of each evaluation factor is normalized to fall within the range of zero to one.

## 4 Data analysis

### 4.1 Construction of pair-wise comparison matrices

The idea of establishing the pairwise comparison matrix of the evaluation index of the barrier-free degree of the station is as follows: the pairwise comparison matrices are constructed between the paired factors under each first-level index to form 3 comparison matrices. Consequently, we engaged 12 experts from various fields related to transit-oriented development (TOD), public space design, and barrier-free accessibility, including architecture, transportation, and user experience design. These experts were responsible solely for evaluating and assigning relative importance. According to Satty, the score is set at 1–9 points, and then the average value is obtained Table 3.

**Table 3:** Comparison matrices of the evaluation framework.

	Accessibility	Continuousness	Functionality	Matching degree
Accessibility	1	3.8	2.2	3.8
Continuousness	/	1	0.625	2.8
Functionality	/	/	1	4
Matching Degree	/	/	/	1

Tables 4–6 showed the comparison matrices of our evaluation framework.

**Table 4:** Comparison matrices of the evaluation framework.

	Material barrier-free facilities	Informational barrier-free facilities
Material barrier-free facilities	1	4
Informational barrier-free facilities	/	1

**Table 5:** Comparison matrices of the evaluation framework.

	Barrier-free elevator	Escalator	Barrier-free stairs and steps	Barrier-free parking lot	Wheelchair ramp	Tactile paving	Barrier-free toilet	Low service desk	Turnstile
Barrier-free elevator	1	2	0.9	3	1.2	0.8	2.4	1	0.8
Escalator	/	1	0.8	2.4	0.9	0.5	1.2	0.7	0.8
Barrier-free stairs and steps	/	/	1	1.8	0.9	0.4333	2.8	1.2	1.8
Barrier-free parking lot	/	/	/	1	0.5	0.4333	1.6	0.5	0.4667
Wheelchair ramp	/	/	/	/	1	0.9	2.6	1	0.9
Tactile paving	/	/	/	/	/	1	3.2	2.4	1.4
Barrier-free toilet	/	/	/	/	/	/	1	0.5	0.333
Low service desk	/	/	/	/	/	/	/	1	1.2
Turnstile	/	/	/	/	/	/	/	/	1

**Table 6:** Comparison matrices of the evaluation framework.

	Visual guide	Braille	Voice prompt	Manual service
Visual guide	1	5	3.8	4.8
Braille	/	1	0.9	1
Voice prompt	/	/	1	1.6
Manual service	/	/	/	1

### 4.2 Weighting calculation

The determination of weightings for the selected assessment factors can be accomplished by utilizing multiple pair-wise comparison matrices. Xue et al. identified four different methods to calculate weighting, namely, geometric average, arithmetic average, eigenvector, and least squares (42). The eigenvector method was employed in this study to calculate the weightings. Finally, the weighting for all the chosen evaluation factors was established, as shown in Table 7. At last, by computing the total rank consistency ratio C.R. < 0.1, the results were considered to be reliable.

**Table 7:** The weight for all the chosen evaluation factors.

Categories	Evaluation factors	Weightings	
Functionality	Tactile paving	0.04	0.30137
	Visual guide	0.0376	
	Turnstile	0.00347	
	Barrier-free elevator	0.0315	
	Wheelchair ramp	0.0301	
	Barrier-free stairs and steps	0.0285	
	Escalator	0.0281	
	Low service desk	0.0261	
	Barrier-free parking lot	0.0235	
	Barrier-free toilet	0.0172	
	Voice prompt	0.0125	
	Braille	0.0125	
	Manual service	0.0103	
Accessibility	Accessibility	0.4066	0.4066
Continuousness	/	0.1606	0.1606
Matching degree	/	0.1012	0.1012

### 4.3 Comprehensive evaluation

The final step explains how to integrate the data that was previously generated to create the final system’s overall score. Each assessment factor’s standardized score from step 4.1 is multiplied by its matching weight from step 4.3, and the sum is added up to produce the comprehensive evaluation index. The evaluation of the barrier-free facilities system in various TOD situations is made more convenient in this way (as shown in Equation 6).


(6) Vaule=ΣB∗W

## 5 Case study

### 5.1 User-centered empirical study

This is an empirical study that selected two metro stations in Shanghai for systematic evaluation and user evaluation and then compared the fitness of the two sets of data. In this study, we combined quantitative methods with user-centered research methods, applying a systematic evaluation framework, questionnaires, and interviews. Though there are various kinds of TOD scenarios, we can only select one scenario of TOD for study due to limited time and resources. Shanghai’s metro system is very typical and has been developed as the highest level in China (29), so the metro scenario has been taken into our study to confirm the practicality of the aforementioned assessment structure. In the end, two stations, namely Xinzhuang station and Xujiahui station, were selected. Since metro Line 1 passed both stations, it would be easier for participants to take part in the tests and for researchers to do the comparison.

### 5.2 Questionnaire and interview

Our questionnaire was modified based on the SUS questionnaire. The System Usability Scale (SUS) is a popular 10-item Likert scale developed by John Brooke in 1989 to evaluate the subjective assessment of usability. The SUS assesses the effectiveness, efficiency, and satisfaction of a system, with participants rating their satisfaction on a scale from one to five (43). The satisfaction index of a questionnaire survey was tested using 10 to 15 questions, which corresponded to the 13 evaluation indices of the evaluation system. The expert prediction method was used to determine the weight of each index. This approach allowed for a quantitative estimation, even in situations where there was limited statistical data or original data available. These results can provide valuable insights and suggestions when such information is not readily accessible.

In addition to collecting survey data, we also incorporated qualitative methods in our study by inviting participants to take photos and conducting open-ended individual interviews to gain insight into their feelings and perceptions of the stations.

### 5.3 Participants

A total of 31 individuals with who lived in the Minhang district were recruited for the study. The participants recruited followed this distribution: older adult individuals (*n* = 7), individuals carrying heavy objects (*n* = 5), individuals with color vision deficiencies (color weakness or color blindness) (*n* = 4), blind individuals (*n* = 3), individuals using wheelchairs for mobility (*n* = 6), and individuals pushing strollers (*n* = 5).

### 5.4 Data gathering process

#### 5.4.1 User rating

The participants’ general routine consisted of several steps. Firstly, they took a taxi to Xinzhuang station. Upon arriving, they entered the station through the South opening and met with our team. At Xinzhuang station, they then experienced all of the facilities that researchers assigned to them. Afterward, the researchers requested them to fill in the SUS questionnaire and held an open-ended individual interview to acquire their feedback on the barrier-free facilities at Xinzhuang station. Next, the participants took the Line 1 railroad to Xujiahui station. At Xujiahui station, they went through a similar process of experiencing the assigned instruments, completing the SUS questionnaire, and taking the interview. Before beginning the tests, the participants were required to sign a written consent form. Additionally, the researchers provided them with detailed information about the tests. As a token of appreciation, each participant received a compensation of 200 RMB after completing the test.

To ensure a smooth execution of the tests, two researchers were involved. One researcher stationed at Xinzhuang station greeted the participants, obtained their written consent, provided detailed instructions for the upcoming tests, and distributed the SUS questionnaire when the participants finished their tests. Simultaneously, the other researcher positioned at Xujiahui station welcomed the participants who had completed the experiment at Xinzhuang station and arrived at Xujiahui station via the subway. This researcher reiterated the instructions and distributed the SUS questionnaire upon completion.

#### 5.4.2 Expert rating

On a separate day from the user experiments, the researchers arrived at the two stations carrying measuring instruments and printed rating sheets to conduct expert evaluations. Besides, due to the available maps of Xujiahui and Xinzhuang Station only including accessible elevators and lacking the markings for many other accessibility facilities, researchers need to visit the subway station and annotate the accessibility facilities based on the planar map.

### 5.5 Analysis and results of two stations

**Figure 1:**
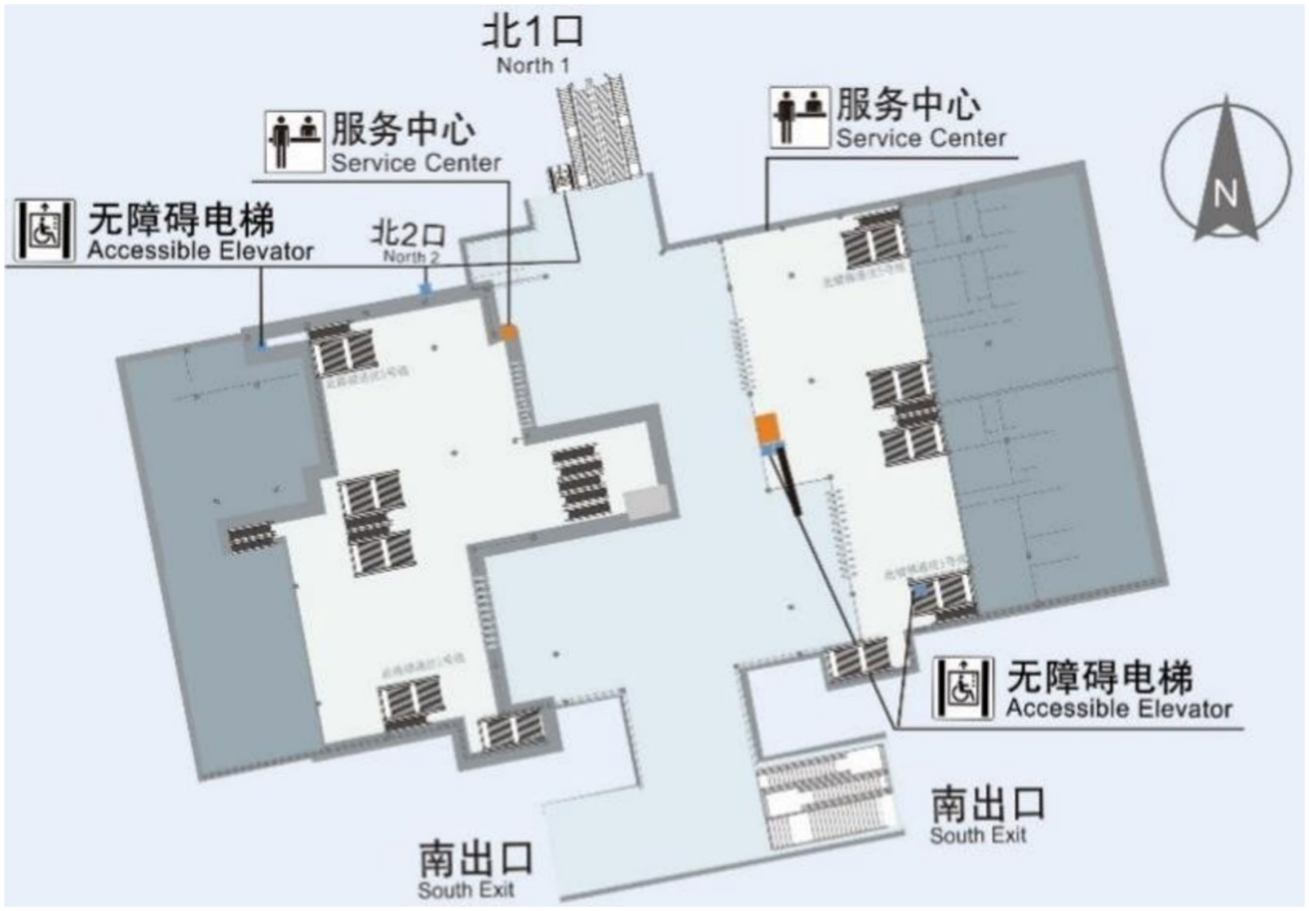
Layout of Xinzhuang station.

**Figure 2:**
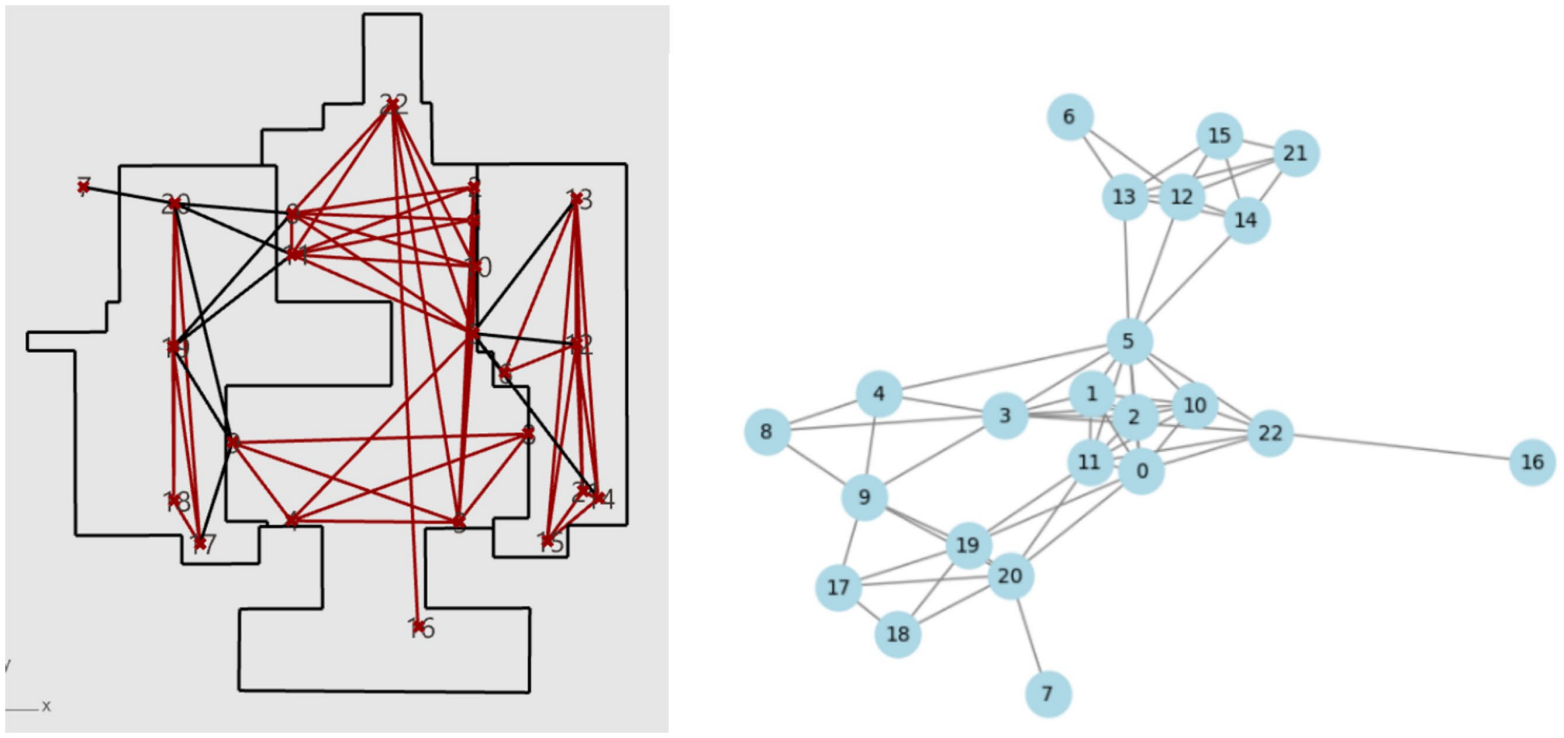
The connection graph of the barrier-free facilities in Xinzhuang station.

**Figure 3:**
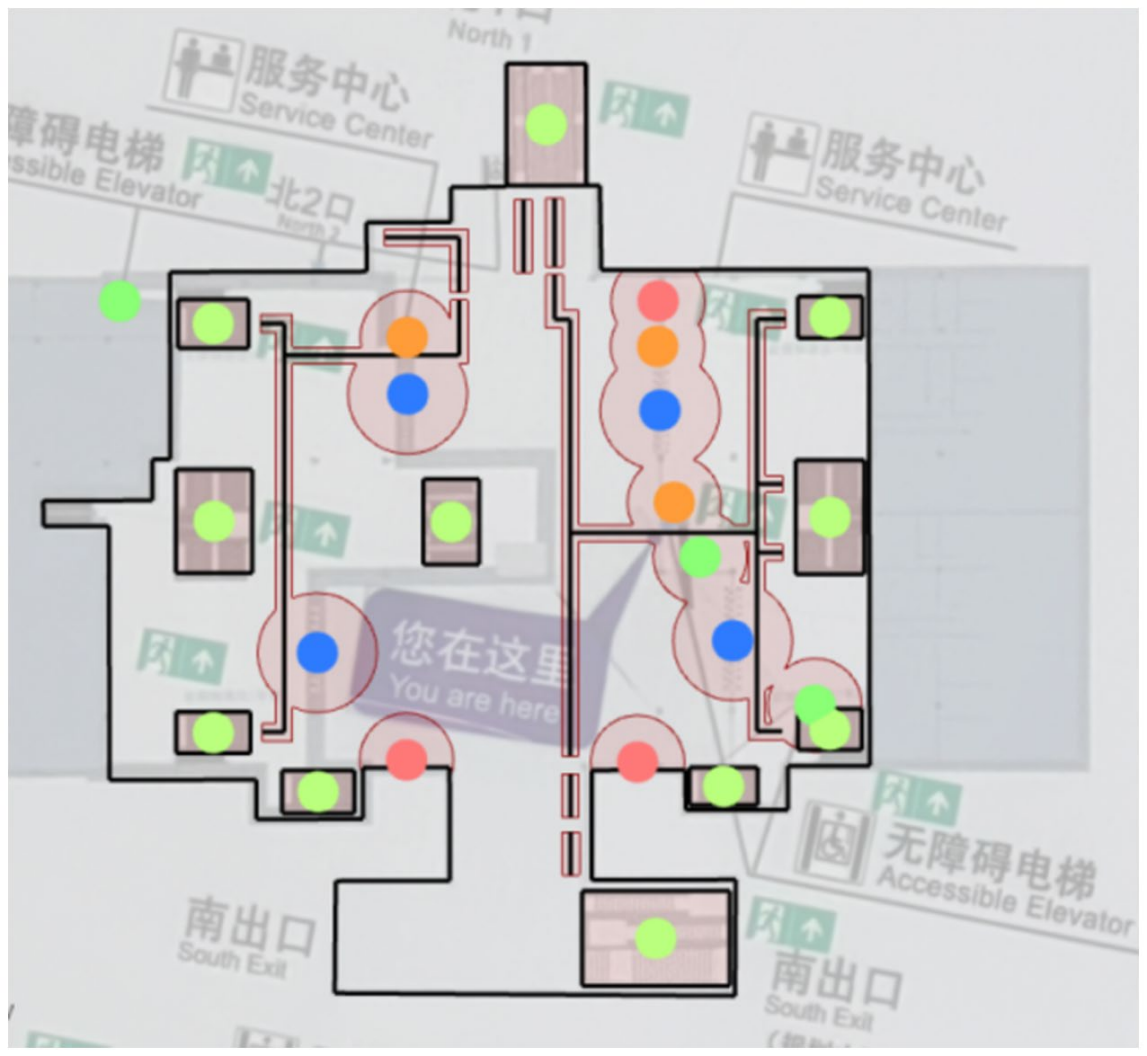
The service area of Xinzhuang station.

**Figure 4:**
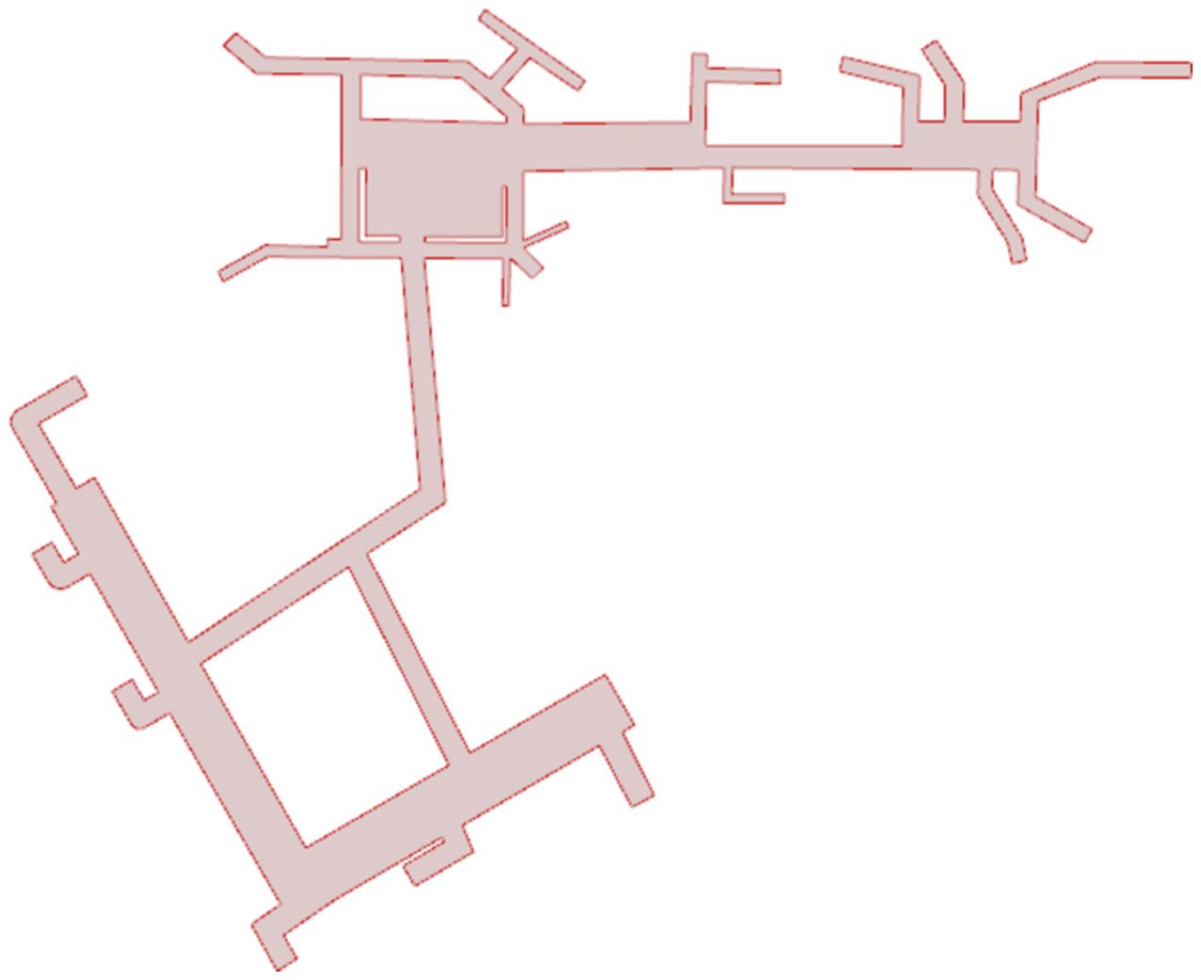
2D flat layout of the Xujiahui station.

Figure 1 shows a planar map of Xinzhuang station. With the annotated information on accessibility facilities, calculations for matching degree and accessibility can be performed. The connectivity between points and the structure of the network, as well as the line connections between these accessibility facilities based on the previously mentioned calculation method, are illustrated in Figure 2. The Connection Graph of the Barrier-free Facilities in Xinzhuang Station. The calculated network depth result is 2.35. Figure 3 shows the service area of Xinzhuang station. And the result of the matching degree is 0.37. The result of continuousness is 0.10. The overall score is 1.19. Figure 4 shows the 2D flat layout of the Xujiahui station. After the same calculation process carried out in Xinzhuang station, the result of the matching degree is 0.33. The result of continuity is 0.45. The result of accessibility is 2.50. The overall score is 1.33.

**Figure 5:**
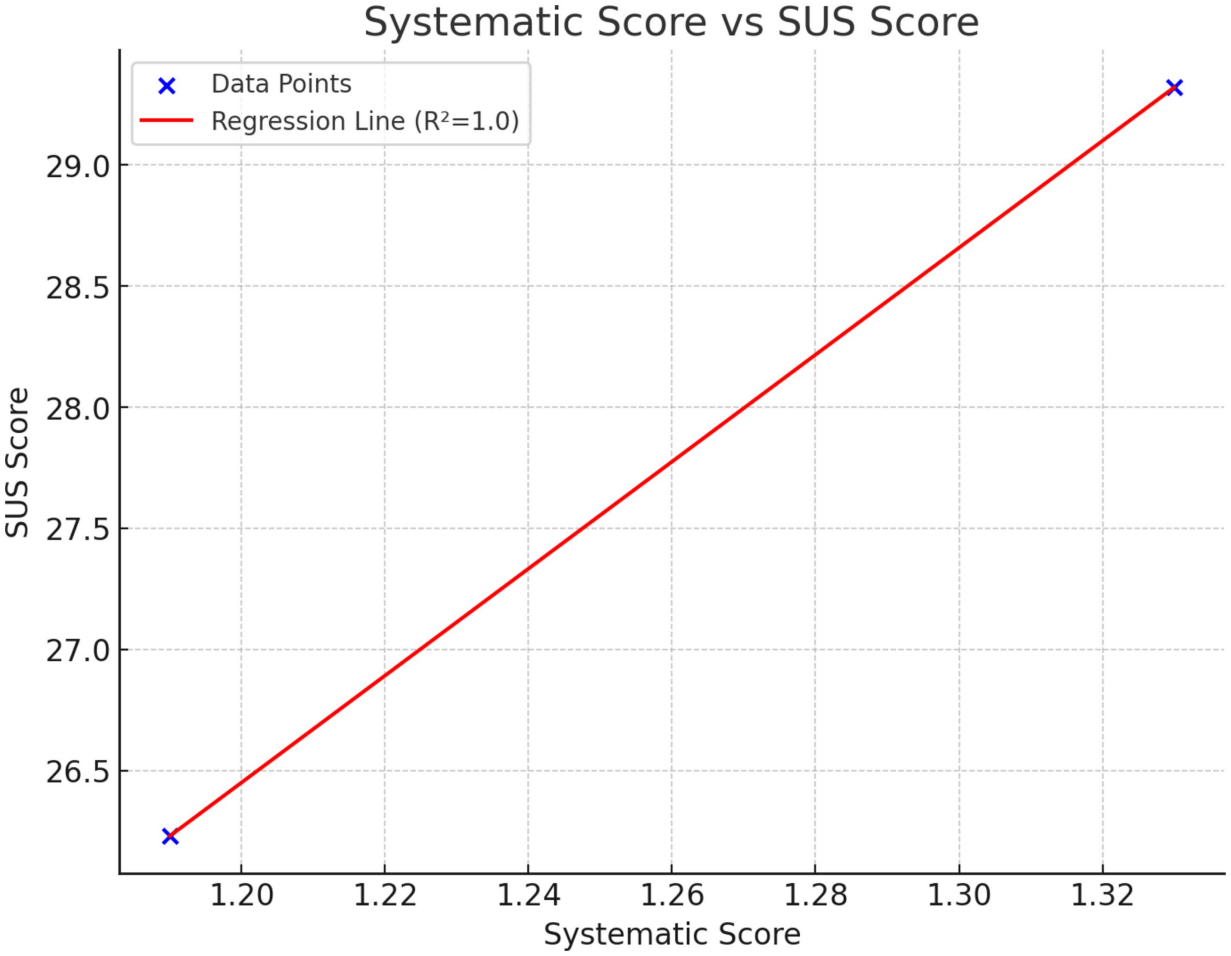
The regression of systemmatic score and SUS score.

The Standard Deviation analysis was conducted to assess the stability of the data. The calculated Standard Deviation value of 0.07 in Xinzhuang station and 2.43 in Xujiahui station suggests that the data is stable. With a low standard deviation, the data points are closely grouped around the mean, indicating minimal variability or dispersion. This implies a consistent and predictable pattern within the dataset, providing confidence in the reliability of the data. Table 8 and Figure 5 shows the systematic score and average SUS score of these two stations. The analysis demonstrates a strong positive correlation between the SUS Score and the expert-assigned Systematic Score, indicating that these two metrics are highly aligned in their assessment of the subject. The Pearson correlation coefficient approaches 1.0, signifying a strong positive linear relationship between SUS Scores and Systematic Scores. The high $R^{2}$ value from the linear regression model further substantiates this relationship, showing that the variations in SUS Scores are largely explained by changes in the expert scores. Additionally, the positive slope of the regression line suggests that higher expert-assigned scores consistently align with higher SUS Scores, underscoring the reliability of expert evaluations as a predictive factor for usability perceptions measured by the SUS. This strong alignment provides robust support for the validity of expert judgments in estimating user satisfaction and usability.

**Table 8:** The systematic scores and average SUS scores.

TOD	Xinzhuang	Xujiahui
Systematic score	1.19	1.33
SUS score (average)	26.23	29.32

**Figure 6:**
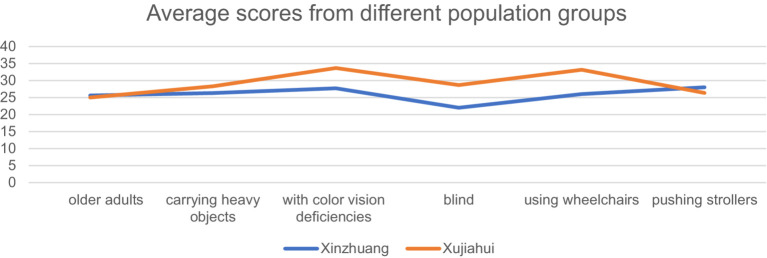
Average scores from different population groups.

As for the SUS score, as shown in Figure 6, the satisfaction level at Xujiahui station is generally higher than that at Xinzhuang station. This indicates that, in general, Xujiahui Station provides a better experience for commuters. The SUS score of Xinzhuang station was scored lower by blind and visually impaired people, as well as color-blind and color-deficient people, and wheelchair users. It indicates that the weak point of Xinzhuang station lies in its wayfinding system. In interviews with users, it was found that the wayfinding system at Xinzhuang station is not very effective in indicating directions and locations of facilities, leading to some participants getting lost during the experimental process. In terms of baby strollers and older adult individuals, Xujiahui station shows a slight weakness, although not significantly. The observed variation may be a result of the constrained sample size. However, the disparity is more pronounced in the case of baby strollers. Based on interviews conducted with individuals related to baby strollers, it has been found that the primary reason behind this discrepancy is the relatively longer routes available at Xujiahui station.

Table 7 showed that the most important factor was accessibility, followed by functionality. In planning barrier-free systems of TOD. Besides, in terms of functionality, the four most important facilities in terms of functionality are tactile paving, visual guide, barrier-free elevators, and wheelchair ramps. Conversely, the three least important facilities are voice prompts, braille, manual services, and turnstile.

Table 9 shows the systematic scores of Xinzhaung station and Xujiahui station. The continuity of Xinzhuang station is particularly much lower than Xujiahui station. Tactile paving, escalators, voice prompts, and wheelchair ramps are also all much lower. Most factors in Xujiahui station are higher than in Xinzhuang. However, the Matching Degree is slightly lower. The main reason is that Xujiahui station is relatively large, and although there are many facilities in this space, it is relatively less dense. In particular, there are few facilities in the strip corridors extending from the central area to the periphery, which lowers the score. In addition, the barrier-free toilet score is also slightly lower for the toilets at Xujiahui station are relatively old. Both Xinzhuang and Xujiahui stations receive a score of zero in terms of barrier-free parking lots, as they both lack designated barrier-free parking lots.

**Table 9:** The systematic scores of Xinzhaung station and Xujiahui station.

Categories	Evaluation factors	Xinzhuang score	Xujiahui score
Functionality	Tactile paving	0.44	0.56
	Visual guide	0.56	0.67
	Turnstile	1	1
	Barrier-free elevator	0.95	0.98
	Wheelchair ramp	0	0.65
	Barrier-free stairs and steps	0.56	0.56
	Escalator	0.33	0.67
	Low service facilities	0.22	0.22
	Barrier-free parking lot	0	**0**
	Barrier-free toilet	1	0.94
	Voice prompt	0.33	0.67
	Braille	0.33	0.33
	Manual service	0.44	0.67
Accessibility	/	2.35	2.5
Continuousness	/	0.1	0.45
Matching degree	/	0.37	0.33

## 6 Discussion

Researchers and practitioners have been committed to developing and implementing reliable assessment methods for accessible design to accurately detect spatial barriers to quality of life, as well as the configuration of various accessible facilities, in order to identify areas that need improvement. At present, there is a lack of clear evaluation criteria for research on accessible facilities, and the study areas are mostly crowded places in campuses and residential areas, without considering areas with high-speed population flow such as TOD. This study is trying to propose an evaluation system to assess the accessibility system of TOD and conducts empirical research to accurately evaluate the quality of accessibility facilities and their impact on urban development.

The study results demonstrate the effectiveness of the proposed evaluation framework for assessing the accessibility of TOD sites in Shanghai. Using the AHP for quantitative assessment and the SUS for user-centered evaluation, consistent findings were observed, confirming the reliability of the evaluation approach. Xinzhuang station received an overall score of 1.19, while Xujiahui station scored 1.33. The higher score for Xujiahui indicates superior accessibility, supported by detailed analysis of individual evaluation factors.

Xujiahui station outperformed Xinzhuang station in accessibility and functionality, with higher scores in key indicators such as tactile paving, visual guidance, and barrier-free elevators. In contrast, Xinzhuang station showed significant deficiencies in the continuity of tactile paving and the availability of wheelchair ramps. Additionally, both stations lacked barrier-free parking facilities, highlighting gaps in accessibility provision.

SUS scores, reflecting user satisfaction with barrier-free facilities, were generally consistent with AHP scores. Xujiahui station received an average SUS score of 29.32, compared to 26.23 for Xinzhuang station. The lower score at Xinzhuang was particularly notable among visually impaired and wheelchair users, who reported difficulties with the wayfinding system and navigation.

Qualitative feedback from participants further supported these findings. Users at Xinzhuang station criticized the ineffectiveness of the visual guidance system, resulting in confusion and challenges in locating accessible routes. A lack of adequate voice prompts and insufficient manual assistance also contributed to lower user satisfaction. In contrast, Xujiahui station’s well-designed wayfinding system and availability of tactile paving were appreciated by users, contributing to its higher SUS score.

A comparative analysis of key evaluation factors revealed important insights into the strengths and weaknesses of each station. Xujiahui’s higher scores for accessibility and functionality were attributed to the availability of tactile paving, well-positioned visual guides, and effective barrier-free elevators. However, Xujiahui’s score for the matching degree—assessing the relationship between user demand and facility supply—was slightly lower due to large spatial areas with fewer accessibility features.

Xinzhuang station exhibited lower continuity scores for tactile paving, negatively affecting overall accessibility. The absence of wheelchair ramps and inadequate manual services further contributed to the station’s lower performance. Nonetheless, Xinzhuang station demonstrated some strengths, such as barrier-free stairs and steps comparable to those at Xujiahui station.

The findings indicate that the proposed evaluation framework effectively identifies the strengths and weaknesses of barrier-free facilities at metro stations. The alignment between AHP and SUS scores suggests that integrating these methods provides a reliable and comprehensive accessibility assessment. Xujiahui station demonstrated superior overall performance, while Xinzhuang station highlighted areas requiring improvement, particularly in wayfinding systems and manual service availability.

The results underscore the importance of incorporating both quantitative metrics and user experiences in evaluating accessibility. By integrating AHP and SUS, the framework ensures that technical performance is complemented by user feedback, offering actionable insights for urban planners and policymakers.

Based on these results, several recommendations can be made to enhance metro station accessibility. For Xinzhuang station, improvements should focus on enhancing the wayfinding system, including upgrading visual guides and adding more voice prompts. Constructing wheelchair ramps and ensuring continuity in tactile paving would significantly enhance accessibility for vulnerable groups. Additionally, improving manual assistance services would increase user satisfaction.

For Xujiahui station, efforts could focus on increasing the density of accessibility features, particularly in peripheral corridors. Updating barrier-free toilets to meet modern standards and considering the installation of moving sidewalks could further enhance accessibility by reducing perceived walking distances. Implementing these recommendations would contribute to a more inclusive and equitable urban transportation environment, supporting the broader goal of sustainable urban development.

## 7 Limitations

The current experiment recruited only 31 qualified participants to evaluate the SUS scores, indicating that the sample size may be insufficient. Additionally, there is a lack of an adequate number of TOD sites to establish the generalizability of this computational tool. Furthermore, human errors are inevitable during the manual data collection process.

### 7.1 Future work

One potential future direction for this research is to propose improvement plans for the identified problems within the barrier-free system of TOD. After implementing these plans, the barrier-free level of the TOD station can be reevaluated and compared with the previous evaluation to determine if any improvements were made. This can provide valuable insights into the effectiveness of the proposed evaluation framework and improvement plans. To carry out this future work, a follow-up study could be conducted after a suitable time interval to collect data and compare the before and after the barrier-free level of the TOD. The data can then be analyzed using statistical methods to determine the significance of any changes. These findings can help improve the design and optimization of barrier-free systems for TOD.

## 8 Conclusion

An evaluation framework for accessibility systems in Transit-Oriented Development (TOD) is essential to ensure accessible use of various public facilities. Although there are standards and studies available for establishing accessibility evaluation structures, existing literature lacks a comprehensive viewpoint.

We try to propose an evaluation framework to assist in the design and optimization of TOD accessibility systems. Through a review of relevant standards and research, 13 quantifiable evaluation factors were identified and standardized. A four-level hierarchical structure was established to assess system performance, and the Analytic Hierarchy Process (AHP) method was used to calculate an integrated evaluation index representing overall performance. The evaluation index generated by this framework can be applied to compare accessibility levels across existing TOD sites.

To demonstrate the feasibility and effectiveness of this evaluation framework, a case study was conducted on two subway stations in Shanghai, China. This case study combined SUS evaluation with user-centered research methods, leading to the following findings:

1. The consistency between user evaluations and system evaluations verified the validity of the system scores. The integration of these two evaluation methods is complementary, providing deeper insights and advancing the field’s exploration.
2. This approach highlighted the strengths and weaknesses of the two subway stations in detail, identified potential issues, and offered recommendations to enhance system performance.

To ensure that TOD truly achieves comprehensive accessibility for all individuals, including vulnerable groups, it is crucial to deeply implement inclusive design principles. Inclusive design aims not only to remove physical barriers and create accessible environments and experiences for everyone but also to uphold the dignity and rights of each individual. TOD serves not only as a transportation hub but also as a catalyst for social integration and inclusion. Optimizing accessibility facilities benefits disadvantaged groups, promotes social harmony, and reflects the human-centered focus and social responsibility in urban planning.

## Data Availability

The raw data supporting the conclusions of this article will be made available by the authors, without undue reservation.
